# Smoking Is Positively Related and Alcohol Consumption Is Negatively Related to an Increased Risk of Meniere’s Disease

**DOI:** 10.3390/jcm11175007

**Published:** 2022-08-26

**Authors:** So Young Kim, Juyong Chung, Dae Myoung Yoo, Mi Jung Kwon, Ji Hee Kim, Joo-Hee Kim, Heejin Kim, Hyo Geun Choi

**Affiliations:** 1 Bundang CHA Medical Center, Department of Otorhinolaryngology-Head and Neck Surgery, CHA University, Seongnam 13488, Korea; 2 Department of Otorhinolaryngology-Head and Neck Surgery, Wonkwang University School of Medicine, Iksan 54538, Korea; 3 Hallym Data Science Laboratory, Hallym University College of Medicine, Anyang 14066, Korea; 4 Department of Pathology, Hallym Sacred Heart Hospital, Hallym University College of Medicine, Anyang 14068, Korea; 5 Department of Neurosurgery, Hallym University College of Medicine, Anyang 14068, Korea; 6 Department of Medicine, Division of Pulmonary, Allergy, and Critical Care Medicine, Hallym Sacred Heart Hospital, Hallym University College of Medicine, Anyang 14068, Korea; 7 Department of Otorhinolaryngology-Head & Neck Surgery, Dongtan Sacred Heart Hospital, Hwaseong 18450, Korea; 8 Department of Otorhinolaryngology-Head and Neck Surgery, Hallym University College of Medicine, Anyang 14068, Korea

**Keywords:** smoking, alcohol, Meniere’s disease, risk factors, cohort studies

## Abstract

A few prior researchers presumed the impacts of smoking and alcohol on the risk of Meniere’s disease (MD). This study investigated the relationship between smoking, alcohol consumption, and obesity with Meniere’s disease in an adult population. The ≥40-year-old population in the Korean National Health Insurance Service-Health Screening Cohort 2002–2019 was analyzed. A total of 15,208 patients with MD were matched with 499,658 comparison participants. The current smoking, alcohol consumption, and past medical histories were collected. Body mass index (BMI) was grouped into underweight, normal, overweight, obese I, and obese II. The odds of histories of smoking and alcohol consumption and the BMI group for MD were analyzed using conditional logistic regression analysis. These associations were further analyzed in subgroups of age, sex, smoking, alcohol consumption, and BMI. In the overall adult population, smoking and alcohol consumption did not show an association with MD. Being underweight was linked with lower odds for MD (adjusted OR [aOR] = 0.80, 95% CI = 0.68–0.93, *p* = 0.004). In the male group, smoking was positively associated with MD (aOR = 1.08, 95% CI = 1.00–1.17, *p* = 0.043), while alcohol consumption was negatively related to MD (aOR = 0.87, 95% CI = 0.81–0.94, *p* < 0.001). Being underweight was related to a lower risk of MD. In adult men, smoking was predicted to increase, while alcohol consumption was predicted to decrease the risk of MD.

## 1. Introduction

Obesity is a common disease whose prevalence is estimated to be approximately 36.9% in Meniere’s disease (MD), a peripheral vestibular disorder that can be diagnosed based on the cochleovestibular symptom complex with a relapsing nature. Its prevalence has been estimated to be approximately 0.043–0.22% in Western countries [1,2] and 0.0058–0.037% in Asian countries [3,4]. Endolymphatic hydrops has been acknowledged as the main pathophysiology of MD [5]. However, various subtypes of MD without endolymphatic hydrops have been introduced [6]. Thus, the etiology of MD is multifactorial. Autoimmunity, inflammation, and cytokines have been reported to induce MD.

For instance, the presence of allergies and immune dysfunction were related to a higher rate of MD (adjusted odds ratio [aOR] = 2.2, 95% CI = 1.8–2.6, *p* < 0.001) [4]. Various treatment strategies have been applied to patients with MD according to the severity and types of disease [7]. The modality of treatment options includes surgeries of endolymphatic sac decompression or vestibular neurectomy, prescription of medicines of betahistine, diuretics, anxiolytics, and lifestyle modifications [7,8,9].

Lifestyle modification can be an initial management option for MD [9]. Due to the recurrent attacks of MD, the management of MD by lifestyle modification can be a cost-effective and noninvasive modality for the treatment and prevention of MD. Thus, lifestyle factors, such as obesity, smoking, and alcohol consumption, have been investigated for their possible impact on MD [4]. A number of previous studies investigated the impacts of dietary modifications of salt, caffeine, and alcohol consumption in patients with MD [10,11]. However, there has been no concordant evidence for the effect of these life factors on MD [10]. This weak evidence can be attributed to the relatively small population and lack of a control population, which can minimize the confounding effects of known risk factors for MD, such as age and sex [4].

This study aimed to estimate the impact of lifestyle factors on the occurrence of MD. To test the associations of a few main lifestyle factors recommended for modification in patients with MD, particularly smoking, alcohol consumption, and obesity, the occurrence of MD was analyzed for the relation to these lifestyle factors. This study is novel in concurrently analyzing a number of lifestyle factors in patients with MD compared with a matched comparison group.

## 2. Materials and Methods

### 2.1. Study Population and Participant Selection

The ethics committee of Hallym University (2019-10-023) permitted the current research. Written informed consent was waived by the Institutional Review Board.

Participants with MD (ICD-10 codes: H810) were enrolled from among 514,866 participants with 895,300,177 medical claim codes from 2002 to 2019 in the Korean National Health Insurance Service-Health Screening Cohort (n = 15,208). The comparison group was enrolled if participants were not diagnosed with MD from 2002–2019 (n = 499,658). The participants who were diagnosed in 2002 were excluded (washout periods, n = 539).

The exclusion criteria were as previously described [12]. In brief, participants who had histories of head trauma, ≥2 times with head and neck CT, brain tumors, disorders of acoustic nerves, and benign neoplasms of cranial nerves were excluded. Patients with MD without a record of body mass were excluded (n = 143). Some of the patients without a record of total cholesterol were excluded (n = 2).

Comparison participants were excluded if they were diagnosed with MD without audiometric examination or diagnosed with MD once (n = 23,239).

MD participants were 1:4 matched with control participants for age, sex, income, and region of residence. The comparison participants were selected in a random number order.

**Figure 1:**
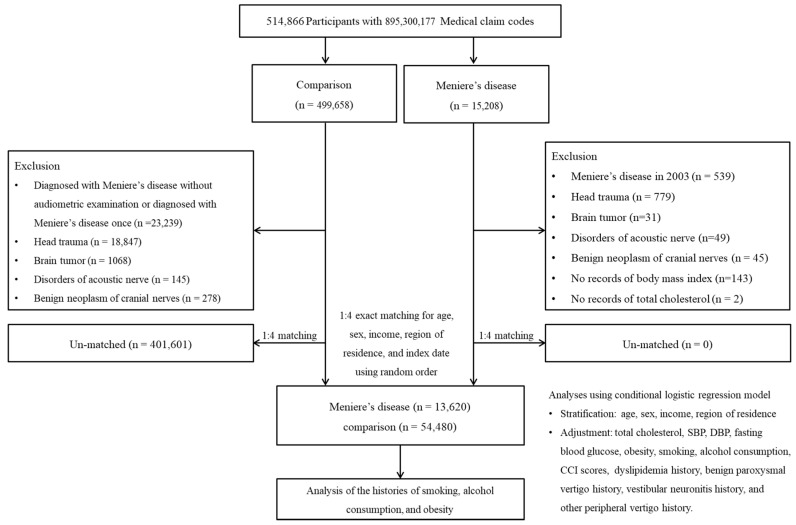
A schematic illustration of the participant selection process used in the present study. Of a total of 514,866 participants, 13,620 MD participants were 1:4 matched with 54,480 comparison participants for age, sex, income, and region of residence. (SBP: systolic blood pressure; DBP: diastolic blood pressure).

The index date of each MD participant was the date of diagnosis of MD. The index date of comparison participants was set as the index date of their matched MD participants. Ultimately, 13,620 MD participants were 1:4 matched with 54,480 comparison participants (Figure 1).

### 2.2. Outcome (MD)

MD was defined if the participants were diagnosed with ICD-10 codes H810 (MD), ≥2 times of treatment histories, and with audiometric examination [12]. The diagnosis of MD was made by a physician, mostly by an otology specialist.

### 2.3. Exposure

The status of smoking was classified as nonsmoker, past smoker, and current smoker based on the self-reported survey questionnaire. The frequency of alcohol consumption was divided into <1 time a week and ≥1 time a week based on the self-reported survey questionnaire. The group of body mass index (BMI, kg/m^2^) was categorized as <18.5 (underweight), ≥18.5 to <23 (normal), ≥23 to <25 (overweight), ≥25 to <30 (obese I), and ≥30 (obese II) based on the Asia-Pacific criteria following the Western Pacific Regional Office (WPRO) 2000 [13].

### 2.4. Covariates

Age was grouped into 10 groups. The levels of income were classified into five groups. The region of residence was divided into urban and rural areas. The blood levels of total cholesterol (mg/dL), systolic blood pressure (SBP, mmHg), diastolic blood pressure (DBP, mmHg), and fasting blood glucose (mg/dL) were collected.

### 2.5. The Charlson Comorbidity Index (CCI) Was Analyzed as a Continuous Variable [14,15]

The prior histories of benign paroxysmal vertigo (BPPV), vestibular neuronitis (VN), other peripheral vertigo, and dyslipidemia were defined based on the ≥2 times of treatment records.

### 2.6. Statistical Analyses

To estimate the odds ratios (ORs) with 95% confidence intervals (CIs) of past medical history for MD, conditional logistic regression analysis was applied. In these analyses, crude (simple) and adjusted models (age, sex, income, region, CCI, total cholesterol, SBP, DBP, fasting blood glucose, dyslipidemia, BPPV, VN history, and other peripheral vertigo) were used. The standardized difference (sd) was calculated for the general characteristics between the MD and comparison groups.

For the subgroup analyses using conditional logistic regression, we stratified participants by their age and sex. We conducted additional subgroup analyses using unconditional logistic regression according to obesity, smoking, and alcohol consumption. Two-tailed analyses were conducted. The *p*-value < 0.05 was set as the statistical significance. SAS version 9.4 (SAS Institute Inc., Cary, NC, USA) was utilized.

## 3. Results

A total of 12.7% (1727/13,620) of the MD group and 11.9% (6486/54,480) of the comparison group were current smokers (sd = 0.02, Table 1). The 30.2% (4107/13,620) and 32.7% (17,789/54,480) of MD and comparison groups addressed the one or more times a week of alcohol consumption (sd = 0.05). The distribution of BMI scores was different between the two groups (sd = 0.06). The rates of histories of BPPV, VN, and other peripheral vertigo were higher in the MD group than in the comparison group (50.7% vs. 14.8%, 18.8% vs. 3.8%, and 39.2% vs. 11.5%).

Current smoking was associated with higher odds for MD in the crude model (OR = 1.10, 95% CI = 1.03–1.17, *p* = 0.005, Table 2). However, this association was not maintained in the adjusted model (adjusted OR [aOR] = 1.05, 95% CI = 0.98–1.13, *p* = 0.198). On the other hand, a history of alcohol consumption was related to lower odds for MD in the crude model (OR = 0.87, 95% CI = 0.84–0.91, *p* < 0.001). However, this association was also not maintained in the adjusted model (aOR = 0.97, 95% CI = 0.92–1.01, *p* = 0.154). According to the BMI group, being underweight was linked with lower odds for MD in both the crude and adjusted models (aOR = 0.80, 95% CI = 0.68–0.93, *p* = 0.004).

The age and sex groups were analyzed for the association of smoking, alcohol consumption, and obesity with MD (Table 3). A history of smoking was associated with higher odds of MD in the male group (aOR = 1.08, 95% CI = 1.00–1.17, *p* = 0.043). On the other hand, a history of alcohol consumption was negatively related to MD in the male group (aOR = 0.87, 95% CI = 0.81–0.94, *p* < 0.001). The women group did not demonstrate a significant association between MD with smoking or alcohol consumption. However, the women group showed low odds for smoking and high odds for alcohol consumption related to MD, which was the opposite direction from those of the men group. Thus, interaction analyses were conducted between sex and smoking or alcohol. The results indicated the significant interaction between sex and alcohol consumption (Chi-square = 14.249, *p* < 0001). There was no significant interaction between sex and smoking (Chi-square = 3.054, *p* = 0.081). Being underweight was negatively associated with MD in the ≥ 55-year-old and female groups (aOR = 0.82, 95% CI = 0.70–0.97, *p* = 0.017 for the ≥ 55-year-old group and aOR = 0.79, 95% CI = 0.65–0.96, *p* = 0.017 for the female group).

Current smoking and alcohol consumption and the BMI groups were analyzed for the association of smoking, alcohol consumption, and obesity with MD (Table 4). The non or past smoker group demonstrated a negative association of being underweight with MD. The current smoker group showed an association between alcohol consumption and being underweight with MD. The group with ≥1 time a week of alcohol consumption demonstrated a negative relationship between being underweight with MD. According to the BMI groups, only the overweight group described a negative association between alcohol consumption with MD.

## 4. Discussion

This study delineated the relation between being underweight, smoking, and alcohol consumption with the occurrence of MD. Being underweight was associated with a lower risk of MD. Although there was no association between smoking and alcohol consumption with MD in the overall population, these factors were related to MD in the male group. Specifically, smoking was linked with a higher risk of MD, while alcohol consumption was linked to a lower risk of MD. The current study improved previous knowledge on the associated factors of MD by concurrently assessing multiple lifestyle factors, including smoking, alcohol consumption, and obesity. As these are modifiable factors, lifestyle modifications can be clinically valuable management strategies for patients who suffer from MD.

Being underweight was negatively associated with MD in the overall adult population in our cohort. Although there has been no supporting evidence for the association between being underweight and the occurrence of MD, a few previous reports suggested the risk of MD in patients with obesity [4]. The pro-inflammatory conditions and immune dysregulation associated with obesity could contribute to the occurrence of MD. A number of inflammatory cascades, including nuclear factor-kappa B-mediated inflammation and cytokines, such as interleukin-1β and tumor necrosis factor-α, were suggested to play a role in the pathogenesis of MD [16]. These cytokines are involved in obesity-related inflammation and adipocyte dysregulation [17]. Thus, underweight conditions can have a protective effect on MD by decreasing adipocyte-related inflammation. The small proportion of the obese population in our cohort can mitigate the potential association of obesity with MD in this study.

Current smoking was related to a higher risk of MD in the male group in the present study. Ever smokers had a 2.22-fold higher risk of peripheral vestibular disease, including MD [18]. Another case-control study demonstrated a less effective treatment outcome of vertigo in patients who smoked [19]. Smoking can be linked with MD by increasing the risk of cardiovascular disorders. Smoking is a known major risk factor for cardiovascular disorders [20]. Atherosclerotic changes can increase the risk of vestibular compromise by directly or indirectly impacting the feeding vessels of the vestibular organ [21]. Indeed, the patients with carotid plaque demonstrated a higher risk of peripheral vestibular disorder than control participants (adjusted hazard ratio = 4.41, 95% CI = 1.75–11.14) [21]. Moreover, a retrospective study described a higher rate of MD attacks within six months in MD patients with a higher number of cardiovascular comorbidities [22].

On the other hand, a history of alcohol consumption was associated with a lower risk of MD in the male group in this study. This result was somewhat contrary to the routine recommendation by a physician to relieve the symptoms of MD. However, a few previous studies suggested supportive evidence for the inverse association between alcohol consumption and MD. It was supposed that alcohol can delay the onset of MD by reducing vasopressin synthesis [23]. Alcohol inhibited vasopressin synthesis by inhibiting hypothalamic function [24]. Decreased vasopressin can induce diuresis, which can relieve abnormally increased endolymphatic pressure in patients with MD. The infusion of vasopressin induced endolymphatic hydrops in guinea pigs [25]. Indeed, the plasma level of vasopressin was increased in patients with MD [10]. In addition, in patients with intractable MD, plasma vasopressin levels were decreased following endolymphatic sac drainage surgery, which was correlated with recovery from symptoms of MD [26]. In summary, alcohol consumption could be associated with MD via suppression of diuresis and relief of endolymphatic hydrops. As there was a significant interaction between sex and alcohol consumption, the men-specific association with alcohol consumption can influence the relation of alcohol consumption with MD in this study.

This study used a large, nationwide population cohort. A large comparison population can be randomly selected with matching variables of age, sex, income, and region of residence. The history of MD and other morbidities were collected from the health claim codes, which were objectively diagnosed by a physician. However, our cohort data did not include laboratory data, such as vestibular function tests and pure tone audiometry. As the diagnosis of MD can be mixed with other vestibular disorders, the histories of BPPV, VN, and other peripheral vertigo were adjusted in the analyses. In addition, a number of organic diseases, including head trauma, brain tumors, disorders of acoustic nerves, and benign neoplasms of cranial nerves, were excluded. Another shortcoming of the present study was the potential confounders, which were not considered in the analyses. For instance, stress levels and nutritional uptakes of salt and caffeine could influence the risk of MD. Last, the cross-sectional study design limited the causality between MD and these lifestyle factors.

## 5. Conclusions

Being underweight was inversely related to the occurrence of MD. In the male group, smoking was associated with a higher risk of MD. On the other hand, alcohol consumption was likely to be involved in a lower risk of MD. In patients with MD, lifestyle modification can be recommended for the cessation of smoking.

## Figures and Tables

**Table 1:** General Characteristics of Participants. *BMI* body mass index, kg/m^2^.

Characteristics	Total Participants		
	Meniere’s Disease	Comparison	Standardized Difference
Age (years old, n, %)			0.00
40–44	128 (0.9)	512 (0.9)	
45–49	563 (4.1)	2252 (4.1)	
50–54	1360 (10.0)	5440 (10.0)	
55–59	2370 (17.4)	9480 (17.4)	
60–64	2310 (17.0)	9240 (17.0)	
65–69	2309 (17.0)	9236 (17.0)	
70–74	2089 (15.3)	8356 (15.3)	
75–79	1527 (11.2)	6108 (11.2)	
80–84	728 (5.4)	2912 (5.4)	
85+	236 (1.7)	944 (1.7)	
Sex (n, %)			0.00
Male	4974 (36.5)	19,896 (36.5)	
Female	8646 (63.5)	34,584 (63.5)	
Income (n, %)			0.00
1 (lowest)	2327 (17.1)	9308 (17.1)	
2	1632 (12.0)	6528 (12.0)	
3	2058 (15.1)	8232 (15.1)	
4	2911 (21.4)	11,644 (21.4)	
5 (highest)	4692 (34.5)	18,768 (34.5)	
Region of residence (n, %)			0.00
Urban	5621 (41.3)	22,484 (41.3)	
Rural	7999 (58.7)	31,996 (58.7)	
CCI score (score) (n, %)			0.12
0	7495 (55.0)	33,015 (60.6)	
1	2749 (20.2)	967 (16.8)	
2	1543 (11.3)	5377 (9.9)	
3	815 (6.0)	2851 (5.2)	
≥4	1018 (7.5)	4070 (7.5)	
Obesity (BMI, kg/m^2^, n, %)			0.06
<18.5 (underweight)	254 (1.9)	1394 (2.6)	
≥18.5 to <23 (normal)	4571 (33.6)	18,936 (34.8)	
≥23 to <25 (overweight)	3784 (27.8)	14,687 (27.0)	
≥25 to <30 (obese I)	4553 (33.4)	17,533 (32.2)	
≥30 (obese II)	458 (3.4)	1930 (3.5)	
Smoking status (n, %)			0.02
Nonsmoker or Past smoker	11,893 (87.3)	47,994 (88.1)	
Current smoker	1727 (12.7)	6486 (11.9)	
Alcohol consumption (n, %)			0.05
<1 time a week	9513 (69.9)	36,691 (67.4)	
≥1 time a week	4107 (30.2)	17,789 (32.7)	
Dyslipidemia (n, %)	9078 (66.7)	30,839 (56.6)	0.21
Total cholesterol (mg/dL, mean, SD)	198.3 (38.8)	198.7 (39.4)	0.01
Systolic blood pressure (mmHg, mean, SD)	126.4 (15.8)	127.0 (16.4)	0.04
Diastolic blood pressure (mmHg, mean, SD)	77.3 (10.1)	77.6 (10.4)	0.03
Fasting blood sugar (mg/dL, mean, SD)	100.8 (25.0)	101.9 (28.7)	0.04
Benign paroxysmal vertigo (n, %)	6899 (50.7)	8078 (14.8)	0.83
Vestibular neuronitis (n, %)	2564 (18.8)	2050 (3.8)	0.49
Other peripheral vertigo (n, %)	5340 (39.2)	6257 (11.5)	0.67

Abbreviations: CCI, Charlson comorbidity index. SD, standard deviation. BMI, body mass index.

**Table 2:** Crude and adjusted odd ratios (95% confidence intervals) of smoking, alcohol consumption, and obesity for Meniere’s disease. * Conditional logistic regression analysis, significance at *p* < 0.05. † Stratified model for age, sex, income, and region of residence. ‡ Adjusted model for Charlson comorbidity index, obesity, smoking state (current smoker compared to a non-smoker or past smoker), frequency of alcohol consumption (≥1 time a week compared to <1 time a week), SBP, DBP, fasting blood glucose, total cholesterol, dyslipidemia, benign paroxysmal vertigo, vestibular neuronitis, and other peripheral vertigo.

Characteristics	N of Meniere’s Disease	N of Comparison	Odd Ratios for Meniere’s Disease (95% Confidence Interval)			
	(Exposure/Total, %)	(Exposure/Total, %)	Crude †	*p*-Value	Adjusted †,‡	*p*-Value
Smoking status	1727/13,620 (12.7)	6486/54,480 (11.9)	1.10 (1.03–1.17)	0.005 *	1.05 (0.98–1.13)	0.198
Alcohol consumption	4107/13,620 (30.2)	17,789/54,480 (32.7)	0.87 (0.84–0.91)	<0.001 *	0.97 (0.92–1.01)	0.154
Obesity				<0.001 *		0.038 *
<18.5 (underweight)	254/13,620 (1.9)	1394/54,480 (2.6)	0.75 (0.66–0.86)	<0.001 *	0.80 (0.68–0.93)	0.004 *
≥18.5 to 23 (normal)	4571/13,620 (33.6)	18,936/54,480 (34.8)	1.00		1.00	
≥23 to 25 (overweight)	3784/13,620 (27.8)	14,687/54,480 (27.0)	1.07 (1.02–1.12)	0.007 *	1.01 (0.96–1.07)	0.642
≥25 to 30 (obese I)	4553/13,620 (33.4)	17,533/54,480 (32.2)	1.08 (1.03–1.13)	0.002 *	1.00 (0.95–1.06)	0.963
≥30 (obese II)	458/13,620 (3.4)	1930/54,480 (3.5)	0.98 (0.88–1.10)	0.771	0.94 (0.83–1.06)	0.316

**Table 3:** Crude and adjusted odd ratios (95% confidence interval) of smoking, alcohol consumption, and obesity for Meniere’s disease in each stratified group according to age and sex. * Conditional logistic regression analysis, significance at *p* < 0.05. † Stratified model for age, sex, income, and region of residence. ‡ Adjusted model for Charlson comorbidity index, obesity, smoking state (current smoker compared to a non-smoker or past smoker), frequency of alcohol consumption (≥1 time a week compared to <1 time a week), SBP, DBP, fasting blood glucose, total cholesterol, dyslipidemia, benign paroxysmal vertigo, vestibular neuronitis, and other peripheral vertigo. BMI: body mass index.

Characteristics	N of Meniere’s Disease	N of Comparison	ORs of Meniere’s Disease			
	(Exposure/Total, %)	(Exposure/Total, %)	Crude †	*p*-Value	Adjusted †‡	*p*-Value
**<55 years old (n = 49,270)**						
Smoking	191/2051 (9.3)	715/8204 (8.7)	1.09 (0.91–1.31)	0.353	0.98 (0.80–1.21)	0.873
Alcohol consumption	550/2051 (26.8)	2455/8204 (29.9)	0.83 (0.74–0.94)	0.003 *	0.93 (0.81–1.06)	0.279
Obesity (BMI, kg/m^2^)				0.003 *		0.093
<18.5 (underweight)	22/2051 (1.1)	166/8204 (2.0)	0.56 (0.35–0.87)	0.011 *	0.62 (0.37–1.02)	0.057
≥18.5 to <23 (normal)	781/2051 (38.1)	3291/8204 (40.1)	1.00		1.00	
≥23 to <25 (overweight)	545/2051 (26.6)	2206/8204 (26.9)	1.04 (0.92–1.18)	0.487	0.98 (0.85–1.13)	0.809
≥25 to <30 (obese I)	638/2051 (31.1)	2327/8204 (28.4)	1.16 (1.03–1.31)	0.013 *	1.09 (0.94–1.26)	0.241
≥30 (obese II)	65/2051 (3.2)	214/8204 (2.6)	1.28 (0.96–1.71)	0.090	1.30 (0.92–1.83)	0.133
**≥55 year old (n = 92,705)**						
Smoking	1536/11,569 (13.3)	5771/46,276 (12.5)	1.10 (1.02–1.18)	0.008 *	1.05 (0.98–1.14)	0.182
Alcohol consumption	3557/11,569 (30.8)	15,334/46,276 (33.1)	0.88 (0.84–0.92)	<0.001 *	0.97 (0.92–1.02)	0.284
Obesity (BMI, kg/m^2^)				<0.001 *		0.050
<18.5 (underweight)	232/11,569 (2.0)	1228/46,276 (2.7)	0.78 (0.67–0.90)	0.001 *	0.82 (0.70–0.97)	0.017 *
≥18.5 to <23 (normal)	3790/11,569 (32.8)	15,645/46,276 (33.8)	1.00		1.00	
≥23 to <25 (overweight)	3239/11,569 (28.0)	12,481/46,276 (27.0)	1.07 (1.02–1.13)	0.009 *	1.02 (0.96–1.08)	0.563
≥25 to <30 (obese I)	3915/11,569 (33.8)	15,206/46,276 (32.9)	1.06 (1.01–1.12)	0.016 *	0.99 (0.93–1.05)	0.700
≥30 (obese II)	393/11,569 (3.4)	1716/46,276 (3.7)	0.95 (0.84–1.06)	0.346	0.90 (0.79–1.03)	0.110
**Men (n = 91,300)**						
Smoking	6193/19,896 (31.1)	1672/4974 (33.6)	1.12 (1.05–1.20)	0.001 *	1.08 (1.00–1.17)	0.043 *
Alcohol consumption	10,935/19,896 (55.0)	2436/4974 (49.0)	0.78 (0.74–0.83)	<0.001 *	0.87 (0.81–0.94)	<0.001 *
Obesity (BMI, kg/m^2^)				0.001 *		0.155
<18.5 (underweight)	494/19,896 (2.5)	89/4974 (1.8)	0.76 (0.60–0.96)	0.020 *	0.81 (0.62–1.05)	0.117
≥18.5 to <23 (normal)	6690/19,896 (33.6)	1582/4974 (31.8)	1.00		1.00	
≥23 to <25 (overweight)	5745/19,896 (28.9)	1488/4974 (29.9)	1.10 (1.01–1.19)	0.022 *	1.06 (0.97–1.16)	0.230
≥25 to <30 (obese I)	6534/19,896 (32.8)	1720/4974 (34.6)	1.12 (1.03–1.21)	0.005 *	1.05 (0.96–1.15)	0.249
≥30 (obese II)	433/19,896 (2.2)	95/4974 (1.9)	0.93 (0.74–1.17)	0.533	0.88 (0.68–1.15)	0.351
**Women (n = 50,675)**						
Smoking	55/8646 (0.6)	293/34,584 (0.9)	0.75 (0.56–1.00)	0.050	0.76 (0.55–1.04)	0.082
Alcohol consumption	1671/8646 (19.3)	6854/34,584 (19.8)	0.97 (0.91–1.03)	0.301	1.07 (1.00–1.14)	0.063
Obesity (BMI, kg/m^2^)				<0.001 *		0.161
<18.5 (underweight)	165/8646 (1.9)	900/34,584 (2.6)	0.75 (0.63–0.89)	0.001 *	0.79 (0.65–0.96)	0.017 *
≥18.5 to <23 (normal)	2989/8646 (34.6)	12,246/34,584 (35.4)	1.00		1.00	
≥23 to <25 (overweight)	2296/8646 (26.6)	8942/345,84 (25.9)	1.05 (0.99–1.12)	0.098	0.99 (0.92–1.06)	0.743
≥25 to <30 (obese I)	2833/8646 (32.8)	10,999/34,584 (31.8)	1.06 (1.00–1.12)	0.063	0.97 (0.91–1.04)	0.393
≥30 (obese II)	363/8646 (4.2)	1497/34,584 (4.3)	1.00 (0.88–1.12)	0.931	0.94 (0.82–1.07)	0.345

BMI: body mass index.

**Table 4:** Crude and adjusted odd ratios (95% confidence interval) of smoking, alcohol consumption, and obesity for Meniere’s disease in each group. * Unconditional logistic regression analysis, significance at *p* < 0.05. † Adjusted model for age, sex, income, region of residence, Charlson comorbidity index, obesity, smoking state (current smoker compared to nonsmoker or past smoker), frequency of alcohol consumption (≥1 time a week compared to <1 time a week), SBP, DBP, fasting blood glucose, total cholesterol, dyslipidemia, benign paroxysmal vertigo, vestibular neuronitis, and other peripheral vertigo.

Characteristics	N of Meniere’s Disease	N of Comparison	ORs of Meniere’s Disease			
	(Exposure/Total, %)	(Exposure/Total, %)	Crude	*p*-Value	Adjusted †	*p*-Value
**Non or past smoker (n =59,887)**						
Alcohol consumption	3114/11,893 (28.6)	13,740/47,994 (26.1)	0.88 (0.85–0.93)	<0.001 *	0.99 (0.93–1.04)	0.580
Obesity (BMI, kg/m^2^)				<0.001 *		0.115
<18.5 (underweight)	232/11,893 (2.0)	1258/47,994 (2.6)	0.78 (0.67–0.90)	0.001 *	0.83 (0.70–0.97)	0.022 *
≥18.5 to <23 (normal)	4041/11,893 (34.0)	16,970/47,994 (35.4)	1.00		1.00	
≥23 to <25 (overweight)	3259/11,893 (27.4)	12,742/47,994 (26.6)	1.07 (1.02–1.13)	0.007 *	1.01 (0.96–1.08)	0.655
≥25 to <30 (obese I)	3940/11,893 (33.1)	15,243/47,994 (31.8)	1.09 (1.03–1.14)	0.001 *	1.01 (0.95–1.07)	0.734
≥30 (obese II)	421/11,893 (3.5)	1781/47,994 (3.7)	0.99 (0.89–1.11)	0.897	0.93 (0.82–1.06)	0.281
**Current smoker (n = 8213)**						
Alcohol consumption	993/1727 (57.5)	4049/6486 (62.4)	0.81 (0.73–0.91)	<0.001 *	0.86 (0.76–0.97)	0.017 *
Obesity (BMI, kg/m^2^)				0.287		0.187
<18.5 (underweight)	22/1727 (1.3)	136/6486 (2.1)	0.60 (0.38–0.95)	0.030 *	0.53 (0.31–0.91)	0.022 *
≥18.5 to <23 (normal)	530/1727 (30.7)	1966/6486 (30.3)	1.00		1.00	
≥23 to <25 (overweight)	525/1727 (30.4)	1945/6486 (30.0)	1.00 (0.87–1.15)	0.986	1.02 (0.87–1.19)	0.830
≥25 to <30 (obese I)	613/1727 (35.5)	2290/6486 (35.3)	0.99 (0.87–1.13)	0.916	0.95 (0.81–1.11)	0.502
≥30 (obese II)	37/1727 (2.1)	149/6486 (2.3)	0.92 (0.64–1.34)	0.666	1.03 (0.67–1.57)	0.901
**Consuming alcohol < 1 time a week (n = 46,204)**						
Smoking	734/9513 (7.7)	2437/36,691 (6.6)	1.18 (1.08–1.28)	<0.001 *	1.06 (0.95–1.18)	0.304
Obesity (BMI, kg/m^2^)				<0.001 *		0.246
<18.5 (underweight)	195/9513 (2.1)	969/36,691 (2.6)	0.81 (0.69–0.95)	0.009 *	0.86 (0.72–1.03)	0.092
≥18.5 to <23 (normal)	3214/9513 (33.8)	12,921/36,691 (35.2)	1.00		1.00	
≥23 to <25 (overweight)	2628/9513 (27.6)	9695/36,691 (26.4)	1.09 (1.03–1.16)	0.004 *	1.03 (0.96–1.10)	0.377
≥25 to <30 (obese I)	3141/9513 (33.0)	11,717/36,691 (31.9)	1.08 (1.02–1.14)	0.008 *	0.99 (0.93–1.06)	0.820
≥30 (obese II)	335/9513 (3.5)	1389/36,691 (3.8)	0.97 (0.86–1.10)	0.630	0.94 (0.81–1.08)	0.373
**Consuming alcohol ≥ 1 time a week (n = 21,896)**						
Smoking	993/4107 (24.2)	4049/17,789 (22.8)	1.08 (1.00–1.17)	0.052	1.06 (0.96–1.17)	0.275
Obesity (BMI, kg/m^2^)				0.002 *		0.069
<18.5 (underweight)	59/4107 (1.4)	425/17,789 (2.4)	0.62 (0.47–0.81)	0.001 *	0.66 (0.49–0.89)	0.007 *
≥18.5 to <23 (normal)	1357/4107 (33.0)	6015/17,789 (33.8)	1.00		1.00	
≥23 to <25 (overweight)	1156/4107 (28.2)	4992/17,789 (28.1)	1.03 (0.94–1.12)	0.556	0.99 (0.90–1.09)	0.804
≥25 to <30 (obese I)	1412/4107 (34.4)	5816/17,789 (32.7)	1.08 (0.99–1.17)	0.082	1.03 (0.94–1.13)	0.533
≥30 (obese II)	123/4107 (3.0)	541/17,789 (3.0)	1.01 (0.82–1.24)	0.941	0.94 (0.75–1.19)	0.618
**Underweight (BMI < 18.5, n = 1648)**						
Smoking	22/254 (8.7)	136/1394 (9.8)	0.88 (0.55–1.41)	0.586	0.70 (0.38–1.27)	0.239
Alcohol consumption	59/254 (23.2)	425/1394 (30.5)	0.69 (0.51–0.94)	0.020 *	0.76 (0.53–1.09)	0.132
**Normal weight (BMI ≥ 18.5 to <23, n = 23,507)**						
Smoking	530/4571 (11.6)	1966/18,936 (10.4)	1.13 (1.02–1.25)	0.017 *	1.13 (0.99–1.29)	0.066
Alcohol consumption	1357/4571 (29.7)	6015/18,936 (31.8)	0.91 (0.85–0.97)	0.001 *	0.98 (0.90–1.07)	0.696
**Overweight (BMI ≥ 23 to <25, n = 18,471)**						
Smoking	525/3784 (13.9)	1945/14,687 (13.2)	1.06 (0.95–1.17)	0.309	1.07 (0.93–1.22)	0.336
Alcohol consumption	1156/3784 (30.6)	4992/9695 (34.0)	0.85 (0.79–0.92)	<0.001 *	0.91 (0.83–1.00)	0.049 *
**Obese I (BMI ≥ 25 to <30, n = 22,086)**						
Smoking	613/4553 (13.5)	2290/17,533 (13.1)	1.04 (0.94–1.14)	0.471	0.97 (0.85–1.10)	0.599
Alcohol consumption	1412/4553 (31.0)	5816/17,533 (33.2)	0.91 (0.84–0.97)	0.006 *	1.01 (0.93–1.11)	0.749
**Obese II (BMI ≥ 30, n = 2388)**						
Smoking	37/458 (8.1)	149/1930 (7.7)	1.05 (0.72–1.53)	0.797	1.32 (0.82–2.12)	0.260
Alcohol consumption	123/458 (26.9)	541/1930 (28.0)	0.94 (0.75–1.19)	0.614 *	1.08 (0.82–1.42)	0.576

## Data Availability

Restrictions apply to the availability of these data. Data were obtained from the Korean National Health Insurance Sharing Service (NHISS) and are available at https://nhiss.nhis.or.kr (accessed on 6 June 2019) with the permission of the NHISS.
